# The Cysteine Protease MaOC1, a Prokaryotic Caspase Homolog, Cleaves the Antitoxin of a Type II Toxin-Antitoxin System

**DOI:** 10.3389/fmicb.2021.635684

**Published:** 2021-02-18

**Authors:** Marina Klemenčič, Ana Halužan Vasle, Marko Dolinar

**Affiliations:** Department of Chemistry and Biochemistry, Faculty of Chemistry and Chemical Technology, University of Ljubljana, Ljubljana, Slovenia

**Keywords:** *Microcystis aeruginosa*, RelE/ParE, ParE/ParD, metacaspase, protease, programmed cell death, regulated cell death, toxin-antitoxin system

## Abstract

The bloom-forming cyanobacterium *Microcystis aeruginosa* is known for its global distribution and for the production of toxic compounds. In the genome of *M. aeruginosa* PCC 7806, we discovered that the gene coding for MaOC1, a caspase homolog protease, is followed by a toxin-antitoxin module, flanked on each side by a direct repeat. We therefore investigated their possible interaction at the protein level. Our results suggest that this module belongs to the ParE/ParD-like superfamily of type II toxin-antitoxin systems. In solution, the antitoxin is predominantly alpha-helical and dimeric. When coexpressed with its cognate toxin and isolated from *Escherichia coli*, it forms a complex, as revealed by light scattering and affinity purification. The active site of the toxin is restricted to the C-terminus of the molecule. Its truncation led to normal cell growth, while the wild-type form prevented bacterial growth in liquid medium. The orthocaspase MaOC1 was able to cleave the antitoxin so that it could no longer block the toxin activity. The most likely target of the protease was the C-terminus of the antitoxin with two sections of basic amino acid residues. *E. coli* cells in which MaOC1 was expressed simultaneously with the toxin-antitoxin pair were unable to grow. In contrast, no effect on cell growth was found when using a proteolytically inactive MaOC1 mutant. We thus present the first case of a cysteine protease that regulates the activity of a toxin-antitoxin module, since all currently known activating proteases are of the serine type.

## Introduction

*Microcystis aeruginosa* is a cyanobacterium that occupies various ecological niches and is characterized by a large genetic diversity between strains (Humbert et al., 2013). To date, 6 complete, 74 scaffold-level, and 40 contig-level genome sequences of *M. aeruginosa* strains have been deposited in the National Center for Biotechnology Information Genome database^1^. Among the genetic elements that enable bacteria to thrive despite environmental perturbations are Toxin-Antitoxin (TA) modules, which play a crucial role in bacterial immunity and adaptation, as recently reviewed by Ramisetty (2020). TA systems were first identified as plasmid addiction modules, exerting post-segregational killing of cells that have not received the plasmid (Tsang, 2017). However, these modules are also common on the chromosomes of most free-living bacteria. They all consist of a toxin that inhibits cell growth and an antitoxin that counteracts the activity of the toxin. The majority of toxins are enzymes that affect various cellular processes, such as peptidoglycan synthesis, translation or DNA replication. Depending on the molecular mechanism of toxin neutralization, the TA systems are currently divided into seven different types (I–VII). In type I and III, the antitoxin molecules are small non-coding RNAs, while in all other types the antitoxin molecules are proteins (Wang et al., 2020).

The type II TA systems, in which both toxin and antitoxin are proteins forming a tight complex, are the most common and best characterized. The Toxin-Antitoxin Database—TADB contains a catalog of over 900 prokaryotic genomes with the identified type II TA systems (Shao et al., 2011). Interestingly, among the organisms listed, the organism with the most TA systems in its genome is *Microcystis aeruginosa* NIES-843, the only *M. aeruginosa* strain in this database. In its 5,842,795 bp large genome, 113 TA systems can be found. In comparison, in a model cyanobacterium *Synechocystis* sp. PCC 6803 with a genome size of 3,573,470 bp, only 19 TA systems were identified (and a further 18 on five of its seven plasmids). Most of the identified type II systems in *M. aeruginosa* can be assigned to the RelBE family, one of the best-documented type II TA systems with detailed reports on its structural and functional properties.

RelE-like toxins belong to the widespread type II RelE/ParE superfamily (Guglielmini and Van Melderen, 2011) and can be subclassified based on their determined/predicted three-dimensional structure and mode of action (Leplae et al., 2011). In this superfamily three functionally distinct families were identified: The RelE family of ribosome-directed endoribonucleases (Neubauer et al., 2009), the ParE family of toxins that inhibit DNA gyrase and DNA replication (Fiebig et al., 2010), and the most recently identified family of DNA nicking endonucleases, with Vp1843 as the only representative characterized so far (Zhang et al., 2017). Within the RelE family, several members (HigA, MqsA, YafQ, YafO, etc.) have been identified in different prokaryotes (Zhang et al., 2020).

Unfortunately, cyanobacteria belong to the bacterial phyla with only a few characterized TA modules. Most of the research was conducted on the model cyanobacterium *Synechocystis* sp. PCC 6803, where several toxins with RNase activity were reported (Ning et al., 2011; Kopfmann et al., 2016; Fei et al., 2018). Two studies were performed with filamentous *Anabaena* PCC 7120: one characterized the HicAB (Potnis et al., 2017) and the other the MazEF system (Ning et al., 2013). Despite the high number of type II TA systems in *M. aeruginosa*, none has been characterized experimentally so far.

Type II systems in *M. aeruginosa* attracted our attention after we observed that in the genome of the PCC 7806 strain a putative RelE/ParE-type TA locus is located adjacent to the gene encoding a protease structurally homologous to a caspase (Klemenčič and Dolinar, 2016). We have previously biochemically characterized this protease and, based on its catalytic mechanism and its presumed evolutionary importance, we have termed it as orthocaspase (*M. aeruginosa* orthocaspase 1, MaOC1) (Klemenčič et al., 2015). Proteases are the key regulatory elements in type II TA systems, which by cleavage of the antitoxin release the toxin and thus render it catalytically active. So far, responsibility for this activity has only been found in proteases of the Lon and ClpP families, as reviewed by Muthuramalingam et al. (2016). Both are serine proteases.

In this study we characterized the first putative *M. aeruginosa* TA pair, composed of a RelE/ParE-like toxin and its native antitoxin (IPF_1065 and IPF_1067, respectively, subsequently shortened as 1065 toxin and 1067 antitoxin). We show that the RelE/ParE-like toxin is toxic to *E. coli* cells and forms a heterotrimer with its antitoxin in a ratio of 1:2 (toxin to antitoxin) in solution. By trimming the toxin in its C-terminal region, toxicity has been eliminated. Furthermore, we show that the orthocaspase MaOC1, a cysteine protease encoded adjacent to the TA pair on the *M. aeruginosa* genome, cleaves the antitoxin in its free form but not when in complex with the toxin, thereby regulating the availability of the toxin in the cell.

## Materials and Methods

### Bacterial Cultures

#### *Escherichia coli* Strains

For cloning of the plasmids, *E. coli* DH5α cells were used (Thermo Fisher Scientific, Waltham, Massachusetts, United States) while for expression of the recombinant proteins we used the BL21(DE3) strain (Novagen, Merck, Darmstadt, Germany).

#### *Microcystis aeruginosa* PCC 7806

The *Microcystis* strain was obtained from the Pasteur Culture collection of Cyanobacteria, where it is kept under the collection number PCC 7806.

### Cloning of the Constructs and the Plasmids Used

All enzymes used for restriction and ligation, the Phusion High-Fidelity DNA polymerase as well as the cloning pJET1.2/blunt vector were from Fermentas (Thermo Fisher Scientific, Waltham, Massachusetts, United States). The expression vector pET28b(+) was from Novagen (Merck, Darmstadt, Germany), while the pSB1C3 vector was obtained from the iGEM registry^2^. All primer sequences are listed in the Supplementary Table S1.

#### pET28_antitoxin

The IPF_1067 gene coding for a ParD-like antitoxin was amplified by colony PCR from chromosomal *M. aeruginosa* PCC 7806 DNA using the primers ipf_1067_F and ipf_1067_R. The resulting approximately 250-bp long PCR product was digested with *Nco*I and *Xho*I and ligated into the plasmid pET28b(+), which was digested with the same restriction enzymes. The recombinant vector led to the expression of a C-terminally His-tagged antitoxin under the control of the IPTG-inducible T7 promoter.

#### pET28_toxin

The IPF_1065 gene coding for a ParE-like toxin was amplified by colony PCR from chromosomal *M. aeruginosa* PCC 7806 DNA using the primers ipf_1065_F and ipf_1065_R. The resulting approximately 350-bp long PCR product was digested with *Nco*I and *Xho*I and ligated into the plasmid pET28b(+), which was digested with the same restriction enzymes. The recombinant vector obtained led to the expression of the C-terminally His-tagged toxin under the control of the IPTG-inducible T7 promoter.

#### pET28_toxin_ΔC

The C-terminally truncated RelE-like toxin (encompassing residues Met1-His84) coding region was amplified by colony PCR from the pET28_toxin vector using the primers ipf_1065_F and ipf_1065_dT_R. The resulting PCR product, approximately 250 bp long, was digested with *Nco*I and *Xho*I and ligated into plasmid pET28b(+) digested with the same restriction enzymes. The obtained vector led to the expression of C-terminally truncated toxin under the control of the IPTG-inducible T7 promoter.

#### pET28_antitoxin_toxin

The genomic locus encoding IPF*_*1065 and IPF*_*1067 proteins was amplified by colony PCR from chromosomal *M. aeruginosa* PCC 7806 DNA using primers ipf_1067_F and ipf_1065_R. The resulting approximately 580 bp long PCR product was digested with *Nco*I and *Xho*I and ligated into plasmid pET28b(+), which was digested with the same restriction enzymes. This setup led to the expression of the antitoxin and the C-terminally His-tagged toxin under the control of the IPTG-inducible T7 promoter.

#### pET28_MaOC1_(WT or C169A)

The construction of C-terminally tagged wild-type MaOC1 or its proteolytically inactive variant with substitution of the active site Cys by Ala residue has already been described (Klemenčič et al., 2015). These plasmids were used for the expression of the two proteins under the control of the IPTG-inducible T7 promoter.

#### pSB1C3_empty

This vector is based on the pSB1C3 plasmid, containing a constitutive strong promoter with a medium-strong ribosome binding site^3^. This plasmid harbors pMB1 origin of replication (expected copy number of 100–300 per cell) and carries a resistance to chloramphenicol and is therefore compatible for co-transformation with the pBR322 ori present in pET28b(+).

#### pSB1C3_antitoxin

The gene coding for the antitoxin was amplified from the pET28_antitoxin vector using the primers ipf_1067_F_*Xba*I and ipf_1067_RoHT_Pst, introducing *Xba*I to the 5’ of the amplicon and His-tag encoding sequence, and a *Pst*I restriction site to the 3’ of the amplicon. After amplification, the PCR product was digested by *Xba*I and *Pst*I, while the pSB1C3_empty plasmid was digested by *Spe*I and *Pst*I restriction enzymes. The ligation product was used to co-transform the cloning strain *E. coli* DH5α as well as the expression strain BL21(DE3) simultaneously with the pET28_toxin plasmid.

#### pSB1C3_antitoxin_toxin

A pSB1C3-derived plasmid was also used for constitutive expression of the antitoxin-toxin operon, which was amplified from the pET28_antitoxin_toxin vector using 1067_F_*Spe*I and 1065_R_*Pst*I primers, introducing *Spe*I on the 5’ of the amplicon and *Pst*I restriction site on the 3’ of the amplicon. After the amplification, the PCR product and the pSB1C3_empty vector were digested by *Spe*I and *Pst*I restriction enzymes and ligated.

### Growth Curves

Overnight cultures of *Escherichia coli* BL21(DE3) cells transformed with appropriate plasmids were diluted to OD_600_ = 0.05 in LB medium containing 50 μg/ml kanamycin and grown in shaking flasks at 37°C. The OD_600_ values were measured using the UV/Vis spectrophotometer, UV-1600PC (VWR, Vienna, Austria) and determined at the indicated times. If necessary, the cultures were diluted prior to measurement so that the maximum optical density measured was below 1.0. When OD_600_ reached values between 0.4 and 0.6, protein expression was induced by the addition of isopropyl 1-thio-β-d-galactopyranoside (IPTG) to 1 mM final concentration. The cultures were grown on an orbital shaker at 37°C and OD_600_ was followed at 1-h intervals up to 8 h after induction.

### Expression and Purification of the 1067 Antitoxin and the 1065–1067 Complex

*Escherichia coli* BL21(DE3) was transformed with the expression plasmids (pET28_antitoxin or pET28_antitoxin_toxin) and cultivated in shaking cultures at 37°C in autoexpression medium containing 50 μg/ml kanamycin for 6 h and then transferred to 16°C, where they continued to grow overnight under shaking (Studier, 2005). The cell pellet collected from 400 ml of the bacterial culture was resuspended in 20 ml resuspension buffer (20 mM HEPES pH 7.5, 500 mM NaCl, 20 mM imidazole) and sonified 5×5 min using 80% power on the UP100H device (Hielscher, Teltow, Germany) on ice. After centrifugation at 20,000×g for 30 min to remove insoluble debris, the supernatant was applied to a Ni-NTA Superflow Cartridge (Qiagen, Hilden, Germany) connected to ÄKTA FPLC system (Cytiva, Marlborough, United States). After washing with the resuspension buffer, the bound proteins were eluted in the same buffer supplemented with 250 mM imidazole. The peak fractions were collected, concentrated to approximately 5 mg/ml using an Amicon filtration unit (Millipore, Burlington, United States) equipped with a 10 kDa cut-off membrane and applied to a Superdex 75 size-exclusion chromatography column (Cytiva, Marlborough, United States) connected to ÄKTA FPLC system. The column was equilibrated in 20 mM HEPES pH 7.5, 150 mM NaCl and a flow rate of 0.5 ml/min was used for the separation of proteins. The peak fractions were collected, aliquoted and stored at −20°C.

### Circular Dichroism Measurements

CD spectra were collected with an Aviv 62DS spectropolarimeter (Aviv Inc., Lakewood, United States) using a 0.1 cm path-length quartz cuvette and averaging three repetitive scans between 260 and 190 nm. Spectra were recorded at 5 μM final protein concentration at 25°C in 20 mM HEPES pH 7.4. The molar ellipticity [θ] was calculated using the theoretical relative molecular mass of the 1067 antitoxin.

### Light Scattering Assay

Size exclusion chromatography was performed on a Superdex 200 Increase size exclusion column (Cytiva, Marlborough, United States) and right-angle light scattering (RALS) and low-angle light scattering (LALS) were measured with an OMNISEC instrument from Malvern Panalytical (Malvern, United Kingdom) with emission at 640 nm. The scattering data were analyzed with the OMNISEC software. Antitoxin and antitoxin-toxin complex samples (loading concentrations of 0.5 mg/ml) were analyzed in 20 mM HEPES pH 7.4, 150 mM NaCl, at room temperature.

### Western Blotting, Immunodetection, and N-Terminal Sequencing

To analyze the positions of cleavage sites in the antitoxin or antitoxin-toxin complex, the proteins were resolved on a 16% Tris-tricine polyacrylamide gel and then blotted onto a PVDF membrane. For immunological detection of recombinant proteins, the membrane was first blocked with 5% milk in PBST for 1 h, followed by incubation with primary rabbit anti-His antibodies (Antibodies-online, 1,000-fold dilution) for 1 h in 1% milk in PBST. The membrane was then thoroughly washed with PBST and secondary goat horseradish peroxidase-conjugated anti-rabbit IgG antibodies (Antibodies-online, 5,000-fold dilution) were added to 1% milk in PBST. The membrane was incubated for 1 h at room temperature. After the second thorough wash, the membrane was developed with Clarity Western ECL substrate (Bio-Rad, Hercules, United States). Chemiluminescence was recorded with the ChemiDoc imager (Bio-Rad, Hercules, United States). For N-terminal sequencing the membrane after the transfer was briefly stained with Coomassie Brilliant Blue R-250, destained and washed with water. The bands of interest were cut out and proteins analyzed on the Procise Protein Sequencing System 492A (PE Applied Biosystems, Foster City, United States).

## Results

### The Putative TA Pair Is Fenced by Two Direct Repeat Regions Harboring Shorter Inverted Repeat Sequences

**FIGURE 1:**
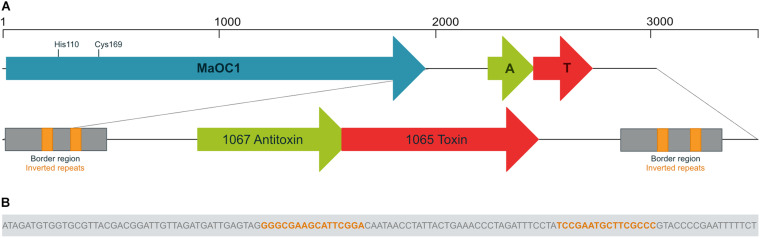
Genomic context of the orthocaspase MaOC1 and the 1065–1067 toxin-antitoxin pair. Genes are shown in reverse orientation compared to the original sequence for clarity. **(A)** In *Microcystis aeruginosa* PCC 7806, the gene encoding the orthocaspase MaOC1 (in blue) is followed by a putative toxin-antitoxin (TA) gene pair (in red and green, respectively). The MaOC1 catalytic dyad, which is formed by His110 and Cys169, is denoted. Downstream and upstream of the TA pair, identical border repeats (in gray) are found, each containing a pair of inverted repeats (in orange). **(B)** The nucleotide sequence of the inverted repeat is shown as part of the border region.

In the annotated genome of *M. aeruginosa* PCC 7806 (Frangeul et al., 2008), the gene encoding the orthocaspase MaOC1 is denoted as IPF_1068 (throughout the article we use locus tags from the EMBL Nucleotide Database). Downstream of it, two partially overlapping protein encoding genes, IPF_1067 and IPF_1065, are present. We previously reported that the first gene encodes a putative antitoxin, while IPF_1065 encodes a toxin belonging to type II group (Klemenčič and Dolinar, 2016). We further analyzed this genomic region and discovered two identical direct repeats flanking the TA module (Figure 1A). The first border region is located 14 base pairs (bp) downstream of the MaOC1 stop codon and ends 147 bp upstream of the Met1 triplet of the putative antitoxin gene. It is repeated in the same orientation 120 bp downstream of the putative toxin stop codon. Each border repeat is 152 bp long and contains a 16 bp long inverted repeat sequence GGGCGAAGCATTCGGA (Figure 1B).

We then performed a BLAST search using the BLASTN algorithm to evaluate the conservation of the nucleotide sequence extending from the beginning of the first border repeat to the end of the second repeat across other bacteria. The results showed the conservation of this sequence with more than 90% only in three other *M. aeruginosa* strains: the analyzed PCC 7806 strain, another PCC 7806 strain designated PCC 7806SL (Zhao et al., 2018) and the recently sequenced FD4 strain (Urakawa et al., 2020). In these strains, the border repeats were conserved and surrounded the TA locus. We then checked for the presence of MaOC1 gene upstream of this sequence. Interestingly, the gene encoding MaOC1 upstream of this TA pair could only be found in PCC 7806 and PCC 7806SL.

### IPF_1065 Encodes a RelE/ParE-Like Toxin

We then compared in detail the nucleotide sequences of the region of interest in the strains PCC 7806 and PCC 7806SL and discovered an 11 bp deletion in the 3’ part of the putative 1065 toxin gene in PCC 7806SL, which leads to a shortening of the C-terminus by 14 amino acid residues and the substitution of 4 amino acid residues at the very C-end of the toxin (Supplementary Figure S1). To determine which of the two PCC 7806 strains we obtained from the Pasteur collection, we sequenced the TA locus and compared its nucleotide sequence with both annotated ones. Our sequence was identical to the PCC 7806SL variant, which is *de facto* the newly sequenced PCC 7806 strain. The nucleotide and translated protein sequences of the TA pair examined in this study are shown in Supplementary Figure S2.

**FIGURE 2:**
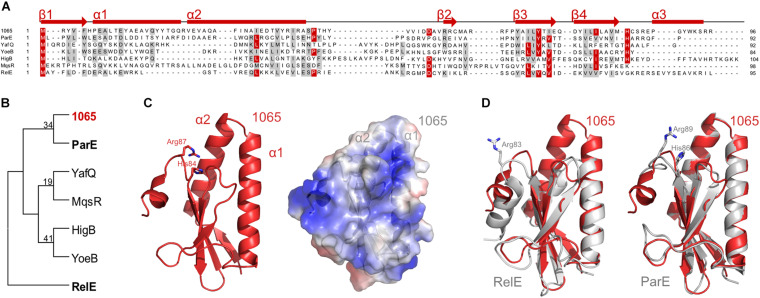
Classification of the 1065 toxin. **(A)** Sequence alignment of the 1065 toxin with representatives of other RelE/ParE toxins (NCBI IDs: ParE, NP_310308; YafQ, NP_414760; YoeB, NP_707909; HigB, NP_311992; MqsR, NP_417494; RelE, NP_416081). Identical residues are colored red and similar amino acids are shaded gray with a threshold of 50% for coloration. Predicted secondary structures of the 1065 toxin are shown above the alignment. The sequence alignment was performed with PROMALS and the image was generated with BioEdit. **(B)** The unrooted phylogenetic tree of selected toxin representatives. The tree was constructed using the Neighbor-Joining method of the MEGA software (version 6) and is based on the alignment shown in panel A. The 1065 toxin is shown in red, while ParE and RelE toxins, whose structures are shown in panel D, are shown in bold. **(C)** Homology model of the 1065 toxin in cartoon representation (left) and its surface potential (right; where red shows the acidic surface potential and blue shows the basic surface potential). The models were predicted by I- TASSER software. **(D)** Overlay of the 1065 toxin model (shown in red) and the RelE (PDB: 4FXE) (left) or ParE (PDB: 5CW7) toxins (shown in gray) (right). Basic amino acid residues known to be responsible for toxic activity are shown as sticks. All images were generated with PyMOL (DeLano Scientific).

To verify the family of type II TA systems to which our pair belongs, we performed a DELTA-BLAST protein homology search, using the 1065 toxin as a search query within the bacterial kingdom. It showed the greatest homology with various type II toxins, which belong to the RelE/ParE family. Primary structures show low conservation between different toxins, as can be seen from the alignment of the 1065 toxin to selected representatives of toxins from this family that have been previously structurally characterized (Figure 2A). All these toxins are derived from *Escherichia coli*, only YoeB is from *Shigella flexneri*.

Based on a phylogenetic analysis, the 1065 toxin showed the closest homology to the *E. coli* ParE protein (Figure 2B). Nevertheless, the overall amino acid sequence conservation was relatively low and, due to very divergent sequences, the bootstrap values are too low to be trusted confidentially. We therefore submitted the amino acid sequence of the 1065 toxin to the I- TASSER server (Yang et al., 2015) to predict its 3D structure, which, in accordance with the DELTA-BLAST analysis, has its highest structural homology with the two available ParE structures [PDB IDs: 5CW7 (Sterckx et al., 2016) and 5CEG (Aakre et al., 2015)]. The 1065 toxin was predicted to fold into a previously characterized RNase fold characteristic of RelE, ParE and YoeB toxins, consisting of two helices folded against a twisted three to four-stranded antiparallel beta sheet (Dalton and Crosson, 2010; Figure 2C). In contrast to RelE, the N-terminal part of the 1065 toxin was predicted to fold into two long alpha helices (α1 and α2), similar to the conformation in ParE (Figure 2D). In addition, the 1065 toxin in the C-terminus harbors two positively charged amino acid residues (His84 and Arg87) that are conserved in most members of the ParE toxin family. However, unlike ParE, it has been predicted that the C-terminus of the 1065 toxin forms a helix similar, if shorter, to that in RelE. Without a reliable tertiary structure and known mode of action, we have not yet been able to assign this toxin to one of the two families, ParE or RelE. For this reason, we continue to treat this protein as the 1065 toxin throughout this paper.

### Toxicity of the 1065 Toxin Is Abolished by Removal of the α3 Helix or in the Presence of the 1067 Antitoxin

**FIGURE 3:**
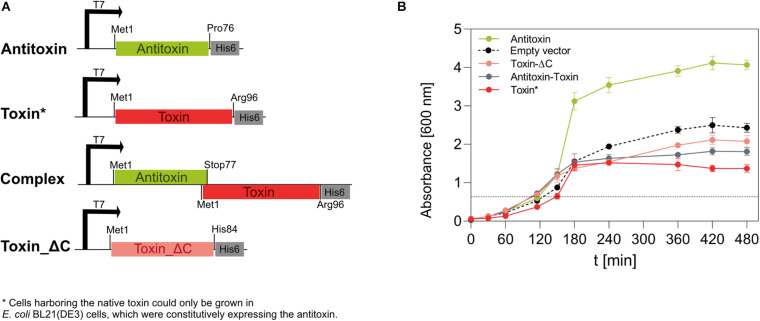
Expression of the 1065 (toxin) and 1067 (antitoxin) genes in *E. coli* BL21(DE3) cells. **(A)** We constructed four pET28b(+)-based vectors that, under the IPTG-inducible T7 promoter, directed the expression of the native antitoxin, the native toxin, the native TA pair (complex) or the C-terminally truncated toxin in *E. coli* BL21(DE3) cells. All genes were inserted in frame with the 3’ His-tag coding sequence. **(B)** The growth of *E. coli* BL21(DE3) cells at 37°C and shaken at 200 rpm was monitored for 480 min by measuring the optical density at 600 nm. The expression of genes under the T7 promoter was induced by 1 mM IPTG when OD_600_ reached about 0.6 (dotted line). Cells containing the pET28_toxin plasmid were co-transformed with the pSB1C3_antitoxin plasmid, allowing constitutive expression of the antitoxin under a strong promoter and a strong ribosome binding site. Cells transformed with the empty pET28b(+) vector were used as controls. Although no gene was under the regulation of the T7 promoter, IPTG was also added at the indicated time point.

We could successfully amplify the IPF_1065 gene and clone it in *E. coli* DH5α in a cloning vector pJET1.2/blunt. However, no *E. coli* cells transformed with the expression vector pET28b(+), which contained the IPF_1065 toxin gene under the inducible T7 promoter, grew, even in the absence of the inducer. Since it has been shown that mutagenesis of the key basic amino acid residues at the C-terminus of toxins of the RelE/ParE family (such as Arg83 in *E. coli* RelE (Pedersen et al., 2002) or complete removal of the C-terminus in ParE (Fiebig et al., 2010) abolished its toxicity, we designed a C-terminally His-tagged truncated variant of the 1065 toxin, comprising only the first 84 amino acid residues (with His84 as the ultimate residue). We named this variant the toxin_ΔC (Figure 3A). *E. coli* BL21(DE3) cells harboring the pET28_ toxin_ΔC plasmid grew normally on both solid and liquid media, even when induced with IPTG, confirming that the toxicity mechanism of the 1065 toxin is similar to that of the RelE/ParE toxins. Although the truncated toxin could be expressed in *E. coli* BL21(DE3) cells, the yields were very low (less than 1 mg/l culture).

Next, we wanted to determine whether IPF_1067 encodes a protein that is able to neutralize the activity of the 1065 toxin. Therefore, we constructed a pSB1C3-based vector, which allowed strong constitutive expression of the antitoxin gene in *E. coli* cells. Only when the cloning DH5α or the expression BL21(DE3) *E. coli* strains were co-transformed with the pSB1C3_antitoxin vector and pET28_toxin vector, colonies developed. When transferred to liquid media, these colonies showed a significantly reduced growth rate even in the absence of the inducer, despite the strongly expressed antitoxin (Figure 3B, red line). When IPTG was added to induce expression of the toxin, cells stopped growing 30 min after induction and their optical density decreased slightly within the next 5 h. The induction of antitoxin expression from pET28_antitoxin had no negative impact on the growth of BL21(DE3) cells (Figure 3B, green line). Moreover, it appeared to visibly increase their growth compared to the induced cells harboring only the empty pET28b(+) vector (Figure 3B, dashed line).

We also expressed the antitoxin-toxin pair from the IPTG-inducible pET28 vector in the arrangement as encoded on the genome of *M. aeruginosa* PCC 7806SL, i.e., starting with the antitoxin Met1 and ending with the wild-type toxin Arg96 in frame with the C-terminal His-tag (Figure 3A, Complex). These cells grew slightly slower in liquid media (Figure 3B, gray line) and reached OD_600_ values slightly lower than cells expressing the non-toxic, truncated toxin variant (Toxin_ΔC).

### The 1067 Antitoxin Is Not Intrinsically Disordered, but Rather Forms a Stably Folded α-Helical Homodimer in the Absence of the Toxin

**FIGURE 4:**
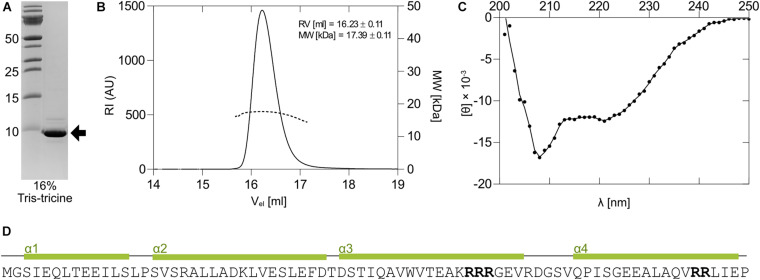
Expression and properties of the 1067 antitoxin. **(A)** The 1067 antitoxin was overexpressed in *E. coli* as a C-terminally His-tagged protein and isolated by nickel-affinity chromatography. It migrated on a 16% Tris-tricine gel with an approximate molecular weight of slightly less than 10 kDa (black arrow). **(B)** Right and Low Light Scattering (RALS/LALS) showed that the antitoxin in solution was mostly present as a molecule with an approximate size of 17.4 kDa, indicating the formation of a dimeric structure. **(C)** The CD spectrum of the 1067 antitoxin in 20 mM HEPES, pH 7.4, showed that at 25°C it exhibited predominantly α-helical structure. This was further confirmed by secondary and tertiary structure prediction models **(D)**, which predicted the folding of the protein into four α-helices. The clusters with two or more Arg residues are marked in bold.

The 1067 antitoxin was readily overexpressed in *E. coli* as a C-terminally His-tagged protein using the pET28_antitoxin plasmid (Figure 3A). While in electrophoresis it migrated as a monomer with the expected molecular weight of about 9.5 kDa on 16% Tris-tricine gel under denaturing conditions (Figure 4A), size exclusion chromatography of the purified antitoxin yielded a single peak on a Superdex 75 10/30 column, but at the elution volume smaller than expected for a monomeric 1067 antitoxin (not shown). To determine its multimeric state, the Right and Low Light Scattering (RALS/LALS) assay was performed. It showed that at room temperature 1067 antitoxin was mostly present as a dimer, which eluted from the Superdex 200 Increase 10/300 column at 16.2 ml, corresponding to an approximate molecular weight of 17.4 ± 0.1 kDa (Figure 4B).

Some antitoxins, especially those belonging to the YefM family, are loosely folded or natively unfolded (Cherny et al., 2005) and acquire their functional fold only upon binding to toxin and/or nucleic acids. However, this does not apply to all type II antitoxins, as some antitoxins of the ParD and YefM families have already been shown to be structured proteins in solution (Kumar et al., 2008; Dalton and Crosson, 2010). We thus performed circular dichroism experiments (CD) to assess the secondary structure of the 1067 antitoxin. The CD spectrum of the antitoxin measured at 25°C showed distinct minima at 208 and 221 nm (Figure 4C), indicating a protein that is largely α-helical with little or no unstructured regions. We also used online available tools to predict the secondary and tertiary structures of the protein (I- TASSER; Yang et al., 2015), which suggested that 1067 antitoxin folds into four α-helices (Figure 4D). Although the 3D structural model confirmed the presence of these helices and even predicted that the four helices would form a bundle, we cannot yet reliably confirm its native structure in solution.

### The 1065 Toxin and 1067 Antitoxin Might Form a Heterotrimer in Solution

Since cells expressing the 1065–1067 TA genomic locus remained viable when induced with IPTG (Figure 3B, Complex), we were able to overexpress and isolate the protein complex via the C-terminal His-tag present on the native toxin (Figure 3A).

**FIGURE 5:**
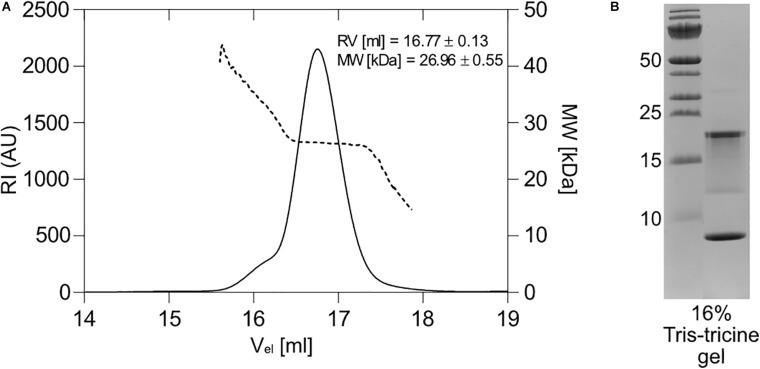
Expression and multimeric form of the 1065–1067 toxin-antitoxin pair. **(A)** Right and Low Light Scattering (RALS/LALS) showed that in solution, the antitoxin-toxin complex was mostly present as a molecule with an approximate size of 27 kDa, indicating the formation of a heterotrimer, in which two molecules of the antitoxin bind to one molecule of the toxin. **(B)** 16% Tris-tricine polyacrylamide gel stained with the Coommasie Brilliant Blue dye. The toxin-antitoxin pair was expressed from the native locus arrangement, but with a His-tag at the C-terminus of the toxin and isolated by nickel affinity chromatography. The expected mass is 12.5 kDa for the His-tag labeled toxin and 8.4 kDa for the antitoxin.

To analyze how the isolated proteins behave in solution under physiological conditions, we applied the purified TA complex on a size-exclusion column and determined its approximate molecular weight using RALS/LALS. The sample was homogeneous and migrated predominantly as a complex with the approximate molecular weight of 27.0 kDa (Figure 5A). Taking into account the dimeric nature of the free antitoxin (the non-tagged antitoxin has a molecular weight of 8.4 kDa) and the presence of the C-terminally tagged toxin (12.5 kDa) in the complex, the formation of a heterotrimer with two antitoxin molecules bound to one toxin is proposed. However, the “shoulder” in the elution diagram at the higher molecular masses suggests the presence of some higher multimeric structures in addition.

When separated by 16% Tris-tricine SDS-PAGE, we expected two bands: one at the antitoxin position (8.4 kDa) and one at the position of the His-tagged toxin (12.5 kDa). In fact, two prominent bands appeared: one below 10 kDa, which we thought was the antitoxin, and one with an approximate mass of about 20 kDa (Figure 5B). A weak distinct band between 10 and 15 kDa was also detected, which corresponds to the expected size of the free His-tagged toxin. Immunodetection with anti-His antibodies revealed that in the range between ∼12 and 22 kDa several His-tagged protein species exist (Supplementary Figure S3B). In order to disprove the possibility that the toxin binds strongly to either a nucleic acid or another entity and thus migrates more slowly, we performed the electrophoretic separation of the complex in 8 M urea 16% Tris-tricine gels using a higher amount of a reducing agent (Supplementary Figure S4A). Although some bands with higher molecular mass disappeared, the ∼20 kDa band remained. Also the smear between 12 and 22 kDa, which is best visible in the immunoblot stain (Supplementary Figure S4B), remained visible despite the presence of urea in SDS-PAGE. Even incubation of the complex with either DNase or RNase did not change the mobility of the 20 kDa band (not shown). To confirm that no extensive insertions/deletions of the antitoxin operon occurred in the BL21(DE3) cells hosting the expression vector, we performed a colony PCR reaction with the sequenced pET28_antitoxin_toxin plasmid as a control (Supplementary Figure S4C). The primers were able to amplify both the antitoxin_toxin locus and the toxin gene itself, suggesting that the genes for the complex are properly present in the *E. coli* expression strain. The anomalous migration of the toxin has yet to be solved in further experiments.

### Orthocaspase MaOC1 Degrades the 1067 Antitoxin in Its Free Form but Not When in Complex With the 1065 Toxin

The levels of free and potentially active toxin in prokaryotic cells are regulated by the action of proteases. So far, only ATP-dependent proteases of the Lon and ClpP families (as reviewed by Muthuramalingam et al., 2016) have been held responsible for antitoxin cleavage. To test whether the orthocaspase MaOC1, which in the genome of *M. aeruginosa* PCC 7806 is encoded upstream of the antitoxin-toxin pair (Figure 1A) can cleave the 1067 antitoxin, we incubated the recombinantly expressed and purified antitoxin either with the wild-type protease or with its proteolytically inactive mutant (substitution of the active site cysteine with the alanine, C169A) and analyzed the reaction mixture by a 16% Tris-tricine SDS-PAGE.

**FIGURE 6:**
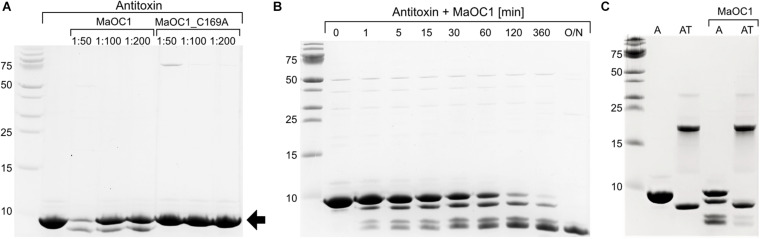
Proteolytic cleavage of the antitoxin or the TA complex with the orthocaspase MaOC1. **(A)** The 1067 antitoxin was incubated in the presence of a wild-type MaOC1 or its proteolytically inactive variant MaOC1_C169A in the mass ratios given for 2 h at room temperature. The reaction mixtures were then electrophoresed on a 16% Tris-tricine gel and stained with Coommasie Brilliant Blue. The arrow represents the expected size of the full-length C-terminally tagged antitoxin (9.5 kDa). **(B)** Reactions containing the antitoxin with MaOC1 (ratio 1:50) were stopped at various times, loaded onto the 16% Tris-tricine gel and stained with Coommasie Brilliant Blue after electrophoresis. **(C)** The antitoxin (A) or the complex (AT) were incubated for 1 h in the absence or presence of MaOC1 orthocaspase (mass ratio 1:100 protein:protease) and applied in duplicates to the 16% Tris-tricine gel. After electrophoresis, the gel was stained with Coommasie Brilliant Blue. All cleavage assays were performed in 20 mM HEPES, pH 7.4, 150 mM NaCl and at room temperature.

While incubation of the MaOC1_C169A mutant with the antitoxin resulted in only one band corresponding to the uncleaved 1067 antitoxin, the wild-type protease cleaved the 1067 antitoxin in a parallel experiment, as shown by the appearance of fragments of lower molecular weight (Figure 6A). The extent of degradation depended on the protease concentration. This suggested that the proteolytic activity of the wild-type protease was indeed responsible for the observed antitoxin degradation. In a time-lapse of the degradation process, a sequential cleavage process was observed, which led to a complete disappearance of the full-length antitoxin and to accumulation of low molecular weight products (Figure 6B). We tried to determine the N-terminal sequences of the three most obvious cleavage products to reveal the exact cleavage sites, but could not obtain any sequences. We explain this with the assumption that all three cleaved protein bands started with Met1 and were therefore C-terminally truncated. Indeed, the inability to obtain sequences could be due to the blocked N-terminus, which is frequently observed in both bacterial and eukaryotic cells (Wellner et al., 1990). C-terminal truncation of the antitoxin was further confirmed by Western blotting, followed by detection of the proteins on the PVDF membrane using anti-His antibodies to detect the tag at the C-terminus of the antitoxin. In fact, only one band could be detected, which most likely represents the antitoxin in full length and therefore not cleaved (Supplementary Figures S3A,B, lanes A MaOC1), while degradation products are not recognized by anti-His antibodies.

To determine whether MaOC1 could also process the TA complex, we repeated the cleavage test with the wild-type protease and the recombinantly expressed and purified TA complex as the putative substrate, with the isolated antitoxin as a control in a parallel experiment (Figure 6C). After 1 h incubation, a visible degradation of the antitoxin was observed, while in the sample containing the complex and the protease only weak additional bands appeared at positions similar to the cleaved antitoxin. No additional cleavage of the antitoxin-toxin complex could be detected even after prolonged incubation (not shown).

### Concomitant Expression of the MaOC1 and 1065–1067 Toxin-Antitoxin Pair Inhibits Growth of *E. coli* Cells *in vivo*

**FIGURE 7:**
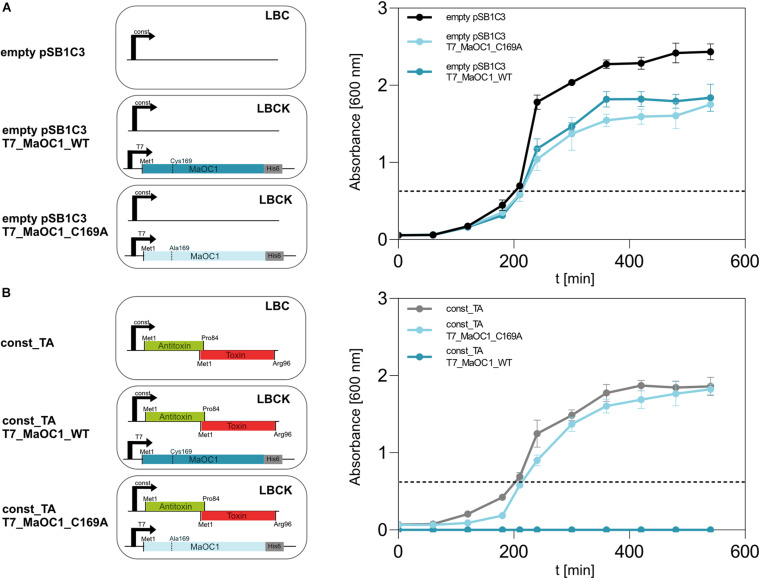
Growth of BL21(DE3) *E. coli* cells expressing TA operon or/and MaOC1 under constitutive and inducible conditions. BL21DE3 *E. coli* cells were transformed either with a pSB1C3_empty **(A)** or a pSB1C3_toxin_antitoxin **(B)** plasmid. Those cells were further used for co-transformation with pET28_MaOC1_WT/C169A plasmid (T7_MaOC1_WT or T7_MaOC1_C169A). The cells containing the respective plasmids were cultured in liquid LB media supplemented with appropriate antibiotics (C, chloramphenicol; K, kanamycin; or both). Overnight cultures diluted to an initial OD_600_ of about 0.05 (except for const_ TA/T7_MaOC1_WT where overnight culture was used) were grown in the respective media at 37°C and OD_600_ was measured at the indicated times. For the expression of genes under the T7 promoter/*lac* operator, IPTG was added to the cultures at 1 mM final concentration (marked with the dotted line).

To assess the dynamics of the interaction between the 1065–1067 TA complex and the protease MaOC1 *in vivo*, we have co-transformed BL21(DE3) *E. coli* cells with two plasmids: pET28b(+) that overexpresses the orthocaspase gene under an IPTG-inducible promoter (T7_MaOC1_WT or its proteolytically inactive C169A variant), and pSB1C3 that encodes the TA operon under a strong constitutive promoter (const_TA). As a control, the same experiment was repeated with an empty pSB1C3 vector harboring the promoter and the ribosome binding site, yet lacking the TA operon (Figure 7).

Based on the observation that the TA operon can be overexpressed in BL21(DE3) cells (Figure 3B), we were interested in phenotypes (survival and growth rates) of cells that constitutively express this operon in the absence or presence (induction with IPTG) of the orthocaspase MaOC1. When BL21(DE3) cells were co-transformed with an empty pSB1C3 vector and either of the two pET28 vectors, viable cells grew in selective liquid media (Figure 7A). However, the co-transformation of the cells with the pSB1C3 vector harboring TA operon the and the pET28 vector harboring wild-type MaOC1, no cells grew even in the absence of an inducer (Figure 7B, dark blue line). On the contrary, cells constitutively expressing the TA operon and harboring the pET28 vector with the proteolytically inactive MaOC1 (pET28_MaOC1_C169A) grew normally on selection plates as well as in liquid media, even when expression of MaOC1_C169 was induced by 1 mM IPTG (Figure 7B, faint blue line).

Our results suggest that overexpression of either the proteolytically active MaOC1 or the TA operon individually does not have a major impact on the survival of recombinant *E. coli* cells (Supplementary Figure S5). However, their simultaneous presence is lethal. Since we have shown by *in vitro* assays that the 1065–1067 TA complex is relatively resilient to MaOC1 proteolysis (Figure 6C), we speculate that MaOC1 targets the 1067 antitoxin primarily during expression and not when in complex with its toxin. Co-translational regulation of the TA pair by proteases instead of cleavage of the antitoxin when in complex with the toxin has already been proposed for other TA systems (LeRoux et al., 2020).

## Discussion

Type II toxin-antitoxin (TA) systems are the most common and best characterized of the six types currently known. They are formed by two protein molecules: a toxin that interferes with essential cellular mechanisms such as DNA replication or protein synthesis, and an antitoxin, which by binding to the toxin forms a tight complex and thus inhibits the toxin. Based on structural similarities, the currently characterized toxins of type II TA systems can be divided into nine superfamilies: RelE/ParE, MazF, HicA, VapC, HipA, FicT/Doc, AtaT/TacT, Zeta, and MbcT (Zhang et al., 2020). However, for a toxin to be functional, it must be released from the complex with the antitoxin. This task is performed in the cells by proteases that specifically cleave the antitoxin, which leads to the activation of the TA module. So far, the only proteases responsible for the degradation of the antitoxin(s) were serine proteases of the Lon and ClpP families (Muthuramalingam et al., 2016).

Interested in the interplay between proteases and TA systems in a bloom forming cyanobacterium *M. aeruginosa*, we first analyzed in detail the genomic region surrounding the only biochemically characterized prokaryotic caspase homolog MaOC1 (Klemenčič et al., 2015) in the *M. aeruginosa* PCC 7806 strain. This strain was recently resequenced and is now labeled PCC 7806SL to distinguish it from the PCC 7806 strain (Zhao et al., 2018).

Downstream of the MaOC1 (IPF_1068, according to the PCC 7806 genome annotation), a putative type II TA locus was identified in which a gene coding for “a plasmid stabilization system protein” (IPF_1065) is preceded by a putative antitoxin gene (IPF_1067), thus forming a canonical bicistronic operon. Since the genes partially overlap, the transcript consists of two overlapping open reading frames (ORFs). This can be observed in most of the type II TA systems, although there are also alternative architectures with the toxin ORF preceding the antitoxin ORF, as shown for mqsRA operon (Brown et al., 2013). Due to the presence of the toxin Shine-Dalgarno sequence within the antitoxin ORF, a “differential translation” has been proposed, which leads to an increased synthesis of an antitoxin compared to the toxin protein per unit of time (Ramisetty, 2020), thus preventing the accumulation of unbound toxin in the cell. According to the amino acid conservation and tertiary structure predictions, IPF_1065 encodes a toxin that belongs to the RelE/ParE superfamily of toxins. This superfamily contains toxins that assume a similar fold, although three different biochemical activities have been described for their members. Toxins of the RelE family (including YafQ and YoeB) inhibit translation by mRNA cleavage (Harms et al., 2018), toxins of the ParE family have been reported to interfere with DNA replication by inhibiting gyrase (Ames et al., 2019), and the *Vibrio parahaemolyticus* toxin Vp1843 has been shown to have a DNA nicking endonuclease activity (Zhang et al., 2017).

Despite the functional differences, the common denominator of toxicity of the RelE/ParE toxins appears to be the C-terminus. Indeed, the mutation of Arg83 in RelE resulted in reduced mRNA cleavage and an almost complete loss of mRNA cleavage was observed in the concomitant mutation of Tyr87 (Neubauer et al., 2009; Griffin et al., 2013). The mutation of His87 in YafQ resulted in the elimination of mRNA cleavage activity and thus loss of toxicity (Prysak et al., 2009). Also in ParE, the deletion of the C-terminus (after His86) led to the loss of its toxicity (Fiebig et al., 2010). Despite the lack of consensus motives, the C-terminus of the 1065 toxin contains positively charged amino acid residues at positions corresponding to those in RelE/ParE toxins. After we removed the C-terminus (after His84), the 1065 toxin lost its toxicity when overexpressed in *E. coli* cells. This suggests that it has a similar molecular mode of action to other members of this toxin superfamily.

We were not able to express the 1065 toxin without coexpressing the 1067 gene, which therefore acted as its antitoxin. The 1067 antitoxin was readily expressed in *E. coli* BL21(DE3) cells and in solution it was mostly present as a dimer. The dimeric nature of antitoxins in solution has already been reported for ParD (Oberer et al., 2007), HigA (Park et al., 2020), and RelB (Li et al., 2008). In contrast to the majority of characterized antitoxins, which are assumed to be intrinsically unstructured proteins, the 1067 antitoxin in solution in the absence of the toxin or DNA showed predominantly α-helical structure and thus showed a similar behavior to the YefM and ParD antitoxins (Kumar et al., 2008; Dalton and Crosson, 2010). Interestingly, the 1067–1065 antitoxin-toxin pair shared another common feature with the YefM antitoxin and its cognate toxin YoeB: They are predominantly present as heterotrimers in solution (Cherny et al., 2005; Kamada and Hanaoka, 2005). Heterotrimeric structures were also reported for RelBE (Overgaard et al., 2008), VapBC (Das et al., 2014), and Phd/Doc (Gazit and Sauer, 1999) type II TA modules.

Regardless of the complex stoichiometry, for each type II TA system the antitoxin must be removed from the complex so that the toxin can become active. This is achieved by the action of proteases and has been studied in great detail in *E. coli*. Among four major proteolytic systems of this bacterium: Lon, ClpP, FtsH, and HsIVU (ClpQY), the first two have been shown to be responsible for antitoxin degradation (Brzozowska and Zielenkiewicz, 2013). Interestingly, the members of the Lon protease family seem to be generally absent in cyanobacteria (Sokolenko et al., 2002; Tripathi and Sowdhamini, 2008), although the gene *sll*0195 of *Synechocystis* sp. PCC 6803, which codes for a protein with 176 amino acid residues and has a 46% similarity to the N-terminal domain of the Lon protease of *E. coli* (785 aa), is involved in the processing of the RelN antitoxin (Ning et al., 2011). No gene coding for a Lon homolog can be found in *M. aeruginosa* PCC 7806.

On the contrary, cyanobacteria contain a rich pool of orthocaspases (Jiang et al., 2010; Asplund-Samuelsson et al., 2012), and the number of orthocaspase genes varies not only from species to species, but can also vary considerably within a species. An illustrative example are different strains of unicellular *M. aeruginosa*, where the number of orthocaspase genes ranges from one (e.g., *M. aeruginosa* PCC 9806, *M. aeruginosa* sp. T1-4), to six orthocaspase genes (*M. aeruginosa* PCC 7806), known as MaOC1-MaOC6 (Klemenčič and Funk, 2018). We have shown that MaOC1 encodes a proteolytically active protease that preferentially cleaves its substrates after clusters of basic amino acid residues (Klemenčič et al., 2015). Interestingly, two such regions can be found in the 1067 antitoxin; the first is located on the putative α3 helix (Lys-Arg-Arg-Arg) and the second on the putative α4 helix (Arg-Arg, Figure 4D). When the 1067 antitoxin was incubated with MaOC1 orthocaspase, specific cleavage was detected at multiple positions. Taking into account their apparent molecular weight and the fact that no C-terminal fragments could be detected with anti-His-specific antibodies (Supplementary Figure S3B), the cleavages most likely occurred at the C-terminus of the antitoxin. The C-termini of the antitoxins are indeed the common targets of Lon and Clp proteases (Brzozowska and Zielenkiewicz, 2013), although this seems to be a consequence of disordered C-terminal structures in the antitoxins, while in 1067 antitoxin the C-terminus most likely adopts a helical fold.

Without detailed knowledge of the 1067 antitoxin structure and the cleavage sites, we are not yet able to explain the molecular mechanisms of this proteolytic interaction. Nevertheless, our results are consistent with previously reported data suggesting that the antitoxins are preferentially cleaved in their free form and not when bound to the toxin and/or nucleic acids. Our *in vitro* and *in vivo* data suggest that MaOC1 targets the 1067 antitoxin when the toxin is not yet bound, i.e., during the process of translation or migration to its final location. This mode of regulation has been proposed previously for ParD-like system Kid/Kis (Diago-Navarro et al., 2013) and for HipAB (Schumacher et al., 2009) TA systems.

While most research now focuses on the cross-regulation between toxin(s) and antitoxins from different systems (Riffaud et al., 2020), little information is available on the interactions between proteolytic systems on the one hand and TA systems on the other. From the study with the Kid/Kis TA module and the proteases Lon, ClpAP, ClpXP, and ClpYQ, where only ClpAP was able to proteolytically process the Kis antitoxin (Diago-Navarro et al., 2013), there seems to be little redundancy between activating proteases.

Cyanobacteria, lacking Lon proteases but containing a rich pool of orthocaspases therefore provide a versatile platform for further studies. This is particularly true for strains of *M. aeruginosa*, which are characterized by high genomic diversity and contain varying numbers of TA modules and orthocaspase-encoding genes. Although *M. aeruginosa* PCC 7806 appears to be the only *Microcystis* strain that contains juxtaposed genes encoding this TA system and this specific orthocaspase, a more detailed bioinformatic analysis involving other TA systems and orthocaspase types could portray a broader picture. In addition, laboratory experiments explaining not only the activation but also the deactivation of the TA systems by proteolysis are needed to explain the cross-regulation between the two regulatory systems.

## Data Availability Statement

All datasets generated for this study are included in the article/Supplementary Material, further inquiries can be directed to the corresponding author/s.

## Author Contributions

MK and MD designed the study. AHV and MK performed the experiments and analyzed the data. MK, AHV, and MD revised the manuscript. All authors contributed to the article and approved the submitted version.

## Conflict of Interest

The authors declare that the research was conducted in the absence of any commercial or financial relationships that could be construed as a potential conflict of interest.

## Footnotes

1 https://www.ncbi.nlm.nih.gov/genome/genomes/820

2 http://parts.igem.org/Part:BBa_K608003

3 http://parts.igem.org/Part:BBa_K608003
