# Supplementary material for: The Cysteine Protease MaOC1, a Prokaryotic Caspase Homolog, Cleaves the Antitoxin of a Type II Toxin-Antitoxin System
Source: Front Microbiol. 2021 Feb 18;12:635684. doi: 10.3389/fmicb.2021.635684 (PMC7935541; doi:10.3389/fmicb.2021.635684)
Supplement: Supplementary file 1 [file Data_Sheet_1.DOCX]

Supplementary Material

# Supplementary Table

**Table S1** **Oligonucleotide primers used in this study.**

All sequences are written in 5′ → 3′ direction. The restriction sites are underlined.

| **Primer name** | **Sequence** |
| --- | --- |
| ipf_1067_F | GACCATGGGGATCAATTGAGCAACTGA |
| ipf_1067_R | TACTCGAGTGGCTCAATCAGTCGTC |
| ipf_1065_F | ATCCCATGGCCAGGTATGTATTTCACCCTGAAGC |
| ipf_1065_R | ATCCTCGAGATCACCGTTAGGCTGC |
| ipf_1065_dT_R | CAGCATCTCGAGATGCATAACCGCCAAAATGAGAATGTAGTC |
| ipf_1067_F_XbaI | TCTAGAATGGGATCAATTGAGCAACTGAC |
| ipf_1067_RoHT_Pst | CTGCAGCTAGTGGTGATGGTGATGATGTGGCTCAATCAGTCGTCTAAC |
| 1067_F_SpeI | TAGTCACTAGTATGGGATCAATTGAGCAACTGAC |
| 1065_R_PstI | GACTACTGCAGCTATCTACGACTTTTCCAGTATCCAGG |

# Supplementary Figures

## Supplementary Figure 1


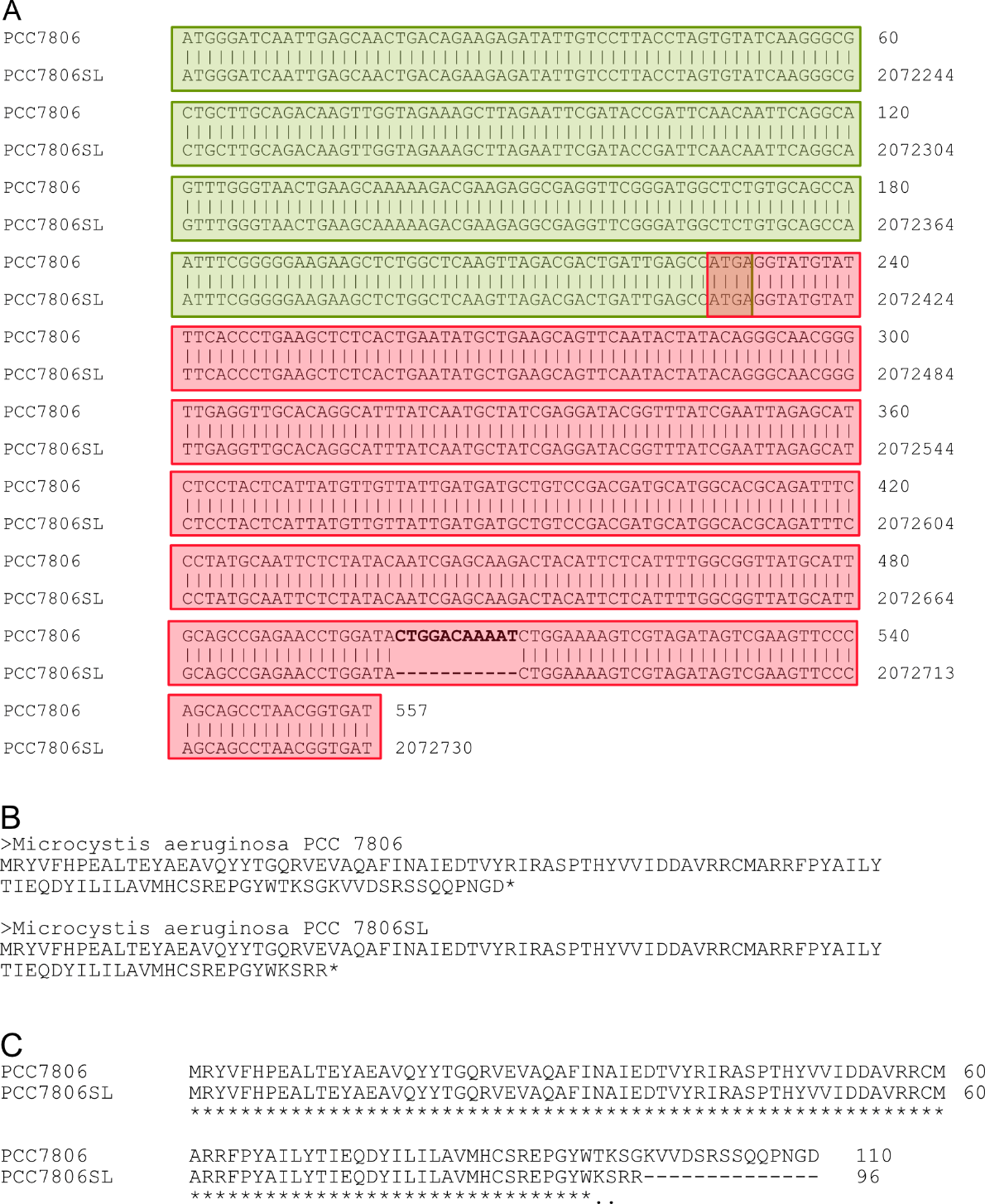


**Fig. S1: Alignment of the nucleotide sequences of the 1067-1065 genomic regions in *M. aeruginosa* PCC 7806 and PCC 7806 SL strains. (A)** Sequence alignment was performed using BLASTN. In green, the nucleotide sequence encoding the 1067 antitoxin and in red the nucleotide sequence encoding the 1065 toxin is shown. Additional 11 bp in the *1065* *toxin* of the PCC 7806 sequence are shown in bold. Sequences and alignment of the translated *M. aeruginosa* PCC 7806 and PCC 7806 SL toxin encoding genes are shown in **(B)** and **(C)**, respectively.

## Supplementary Figure 2


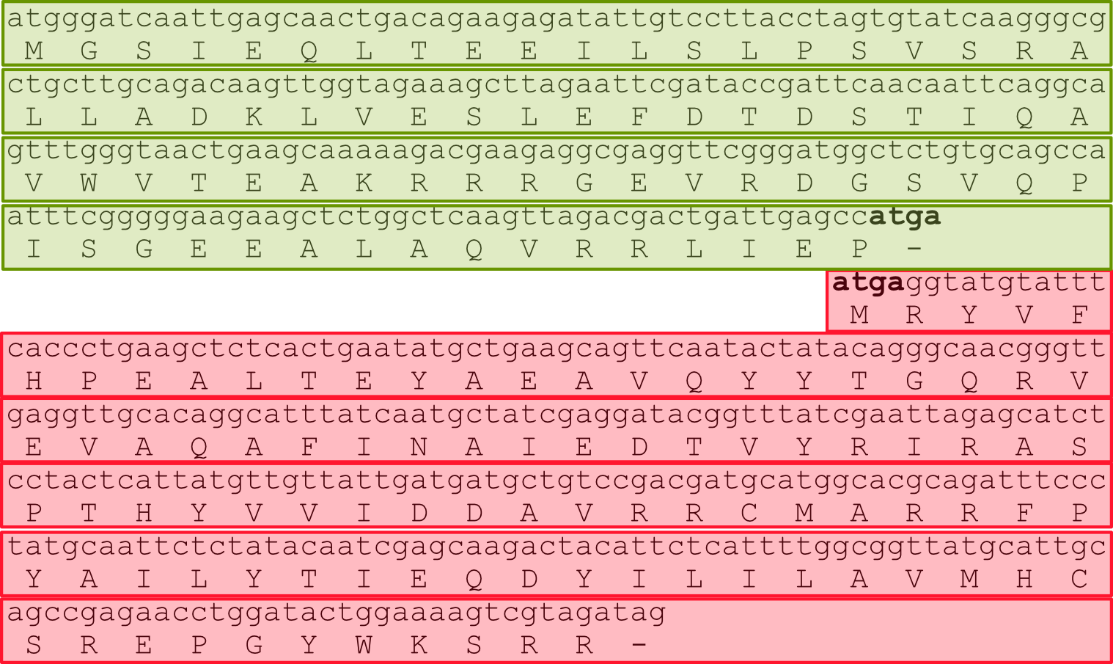


**Fig. S2: Nucleotide and translated protein sequences of the antitoxin 1067 and toxin 1065 used in this study.** The nucleotide sequence and the translated protein sequence of the antitoxin (green) and the toxin (red) used in this study are shown. The four overlapping nucleotides are in bold.

## Supplementary Figure 3


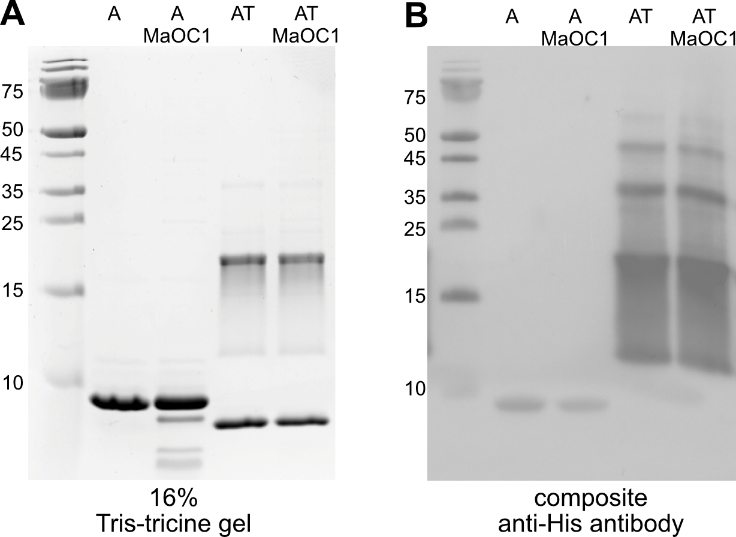


**Fig. S3: Coommasie stain of the 16% Tris-tricine gel and the corresponding PVDF membrane with the detection of the His-tagged proteins using anti-His antibodies.** C-terminally tagged antitoxin (9.5 kDa) or the complex (non-tagged antitoxin, 8.4 kDa and C-terminally His-tagged toxin, 12.5 kDa) were incubated in the absence or in the presence of the MaOC1 orthocaspase (1:100 protein:protease ratio) in 20 mM HEPES, pH 7.4, 150 mM NaCl for 3h and loaded onto the 16% Tris-tricine gel in duplicates. One half of the gel was stained with Coommasie Brilliant Blue (**A**), while the second was transferred to a PVDF membrane for antibody detection using the anti-His primary antibody and HRP conjugated secondary antibody (**B**). Chemiluminiscence of the gel superimposed to the scanned membrane to show the positions of the standards is shown.

## Supplementary Figure 4


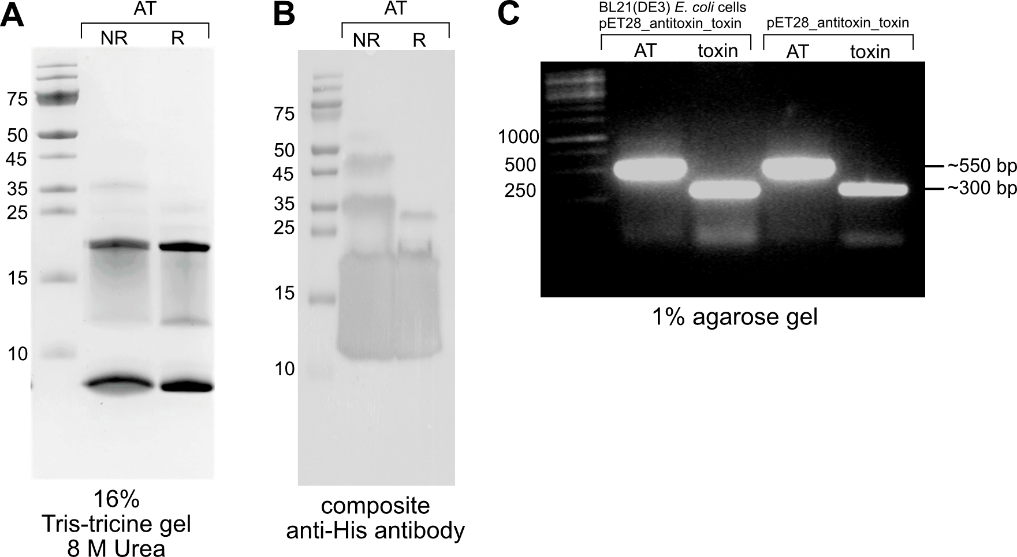


**Fig. S4: Coommasie stain of the 16% Tris-tricine gel in 8M urea, the corresponding PVDF membrane with the detection of the His-tagged proteins using anti-His antibodies and a colony PCR detecting presence of the insert in the BL21DE3 cells harboring pET28_antitoxin_toxin plasmid.** The complex (non-tagged antitoxin, 8.4 kDa and C-terminally His-tagged toxin, 12.5 kDa) loaded onto the 16% Tris-tricine gel containing 8 M urea with the SDS-loading buffer without (NR, non-reducing) or with the reducing agent β-mercaptoethanol (R, reducing) in duplicates. One half of the gel was stained with Coommasie Brilliant Blue (**A**), while the second was transferred to a PVDF membrane for antibody detection using the anti-His primary antibody and HRP conjugated secondary antibody (**B**). Chemiluminiscence of the gel superimposed to the scanned membrane to show the positions of the standards is shown. (**C**) Colony PCR was performed using the BL21(DE3) cells containing the pET28_antitoxin_toxin plasmid to confirm the presence of the antitoxin_toxin operon in the expression strain. The PCR fragments were loaded onto the 1% agarose gel and stained with ethidium bromide. For detection of the whole operon the primers ipf_1067_F and ipf_1065_R were used, while for detection of the toxin within this operon primers ipf_1065_F and ipf_1065_R were used. As a control, the vector pET28_antitoxin_toxin was used. Expected size of the antitoxin_toxin operon is approximately 550 bp, while the toxin is expected to be 330 bp long.

## Supplementary Figure 5


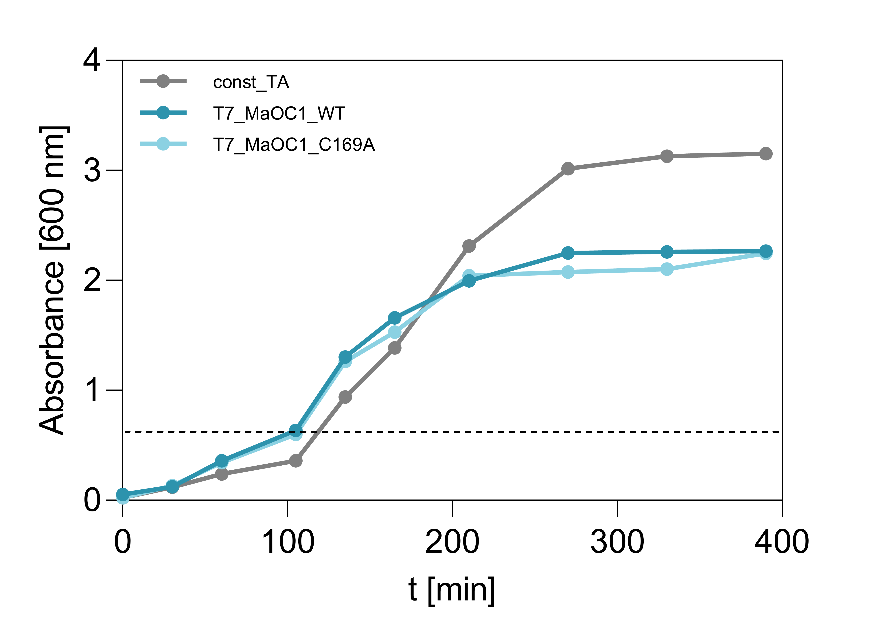


**Fig. S5:** **Growth of BL21(DE3) *E. coli* cells expressing only the TA operon or MaOC1 under constitutive and inducible conditions, respectively.** BL21DE3 *E. coli* cells were transformed with pSB1C3_TA plasmid (const_TA) or the pET28_MaOC1_WT /C169A plasmid. The cells containing the respective plasmids were cultured in liquid LB media supplemented with appropriate antibiotics. Overnight cultures diluted to an initial OD_600_ of about 0.05 were grown in the respective media at 37 °C and OD_600_ was measured at the indicated times. For the expression of genes under the T7 promoter/*lac* operator, IPTG was added to the cultures at 1 mM final concentration (marked with the dotted line).
